# Larvicidal efficacies of plants from Midwestern Brazil: melianodiol from *Guarea kunthiana* as a potential biopesticide against *Aedes aegypti*

**DOI:** 10.1590/0074-02760160134

**Published:** 2016-06-13

**Authors:** Ulana Chaves Sarmento, Carlos Henrique Miguita, Luís Henrique de Oliveira Almeida, Cleusa Rocha Garcia Gaban, Lilliam May Grespan Estodutto da Silva, Albert Schiaveto de Souza, Walmir Silva Garcez, Fernanda Rodrigues Garcez

**Affiliations:** 1 Universidade Federal de Mato Grosso do Sul, Instituto de Química, Campo Grande, MS, Brasil; 2 Universidade Federal de Mato Grosso do Sul, Centro de Ciências Biológicas e da Saúde, Campo Grande, MS, Brasil

**Keywords:** Guarea kunthiana, mosquito control, dengue, Zika, chikungunya, protolimonoid

## Abstract

A total of 36 ethanol extracts from different anatomical parts of 27 plant species (18 families), native to the Pantanal and Cerrado biomes in Midwest Brazil, was assessed for their effect against *Aedes aegypti* larvae, the vector of dengue, hemorrhagic dengue, Zika and chikungunya fevers. Only the extract obtained from seeds of *Guarea kunthiana* (Meliaceae) proved active (LC50 = 169.93 μg/mL). A bioassay-guided investigation of this extract led to the isolation and identification of melianodiol, a protolimonoid, as the active constituent (LC50 = 14.44 *m*g/mL). Meliantriol, which was also obtained from the bioactive fraction, was nevertheless devoid of any larval toxicity, even at the highest concentration tested (LC50 > 100.0 *m*g/mL). These results indicate that the larvicidal activity of melianodiol stems from the presence of the carbonyl moiety at C-3 in the 21,23-epoxy-21,24,25-trihydroxy-tirucall-7-ene-type skeleton. The structures of both protolimonoids were established on the basis of spectral methods (^1^H and ^13^C NMR and MS). This is the first report on the toxicity of melianodiol against *Ae. aegypti* larvae. Based on the results, melianodiol can be regarded as a potential candidate for use as an ecologically sound biocontrol agent for reducing the larval population of this vector.

*Aedes aegypti* is the principal vector of arboviral diseases, such as dengue, hemorrhagic dengue, Zika, chikungunya, and yellow fevers. The incidence of dengue fever has grown remarkably in recent decades worldwide (WHO 2016a). In the American continent, it has increased from 16.4 cases per 100,000 residents in 1980 to 218.3 per 100,000 in the period 2000-2010 and it continues to seriously affect this region. The number of reported cases across South-East Asia, Western Pacific and even in North America and European countries has continued to increase as well, which reveals the threat of outbreaks of dengue fever in these regions (PAHO 2016a, WHO 2016b). In Brazil, from January to December, 2015, 1,649,008 cases were notified (almost three times the number reported for the same period in 2014), including 15,693 severe cases and 886 deaths (PAHO 2016a, WHO 2016b). With regard to 2016 epidemiological update, 224,064 cases of dengue have already been reported in Brazil until the end of February, with 102 severe cases and 52 deaths (PAHO 2016a), therefore warranting urgent measures for its control.

Initially limited to the African continent, Zika fever has expanded its geographical range since 2007. To date, autochthonous Zika virus transmission has been confirmed in a total of 59 countries and territories, with prevalence in those of Latin America and the Caribbean - 31 countries and territories in 2016 (WHO 2016c). This is twice the number that has been reported for this region up to December, 2015, as having locally acquired infection by Zika (PAHO 2016b). According to the World Health Organization, 3 to 4 million infections by Zika virus are estimated in the Americas for 2016, in which 0.4 to 1.3 million cases are anticipated in Brazil, where Zika fever was first confirmed in 2015 (CNN 2016, WHO 2016c). Of greatest concern is a potential established association between Zika virus infection and a growing incidence of microcephaly cases in newborns and neurological disorders, as Guillain-Barré syndrome (GBS). According to Brazilian health authorities, the number of reported cases of congenital microcephaly and/or other central nervous system malformations with laboratory-confirmed Zika virus congenital infection has increased since 2015 (PAHO 2016c). From October 2015 to March 2016, 907 of such cases were confirmed nationwide, while 4,268 cases are still under investigation (PAHO 2016c). Among the 198 child deaths which occurred during pregnancy or after birth, 46 of these were linked to congenital virus infection (PAHO 2016c). In this context, de Noronha et al. (2016) recently provided evidence of human transplacental transmission of Zika virus and also demonstrated its fetal neurotropism. As for the incidence of GBS, 12 countries and territories in the Americas have reported a steep increase in GBS notified cases and/or Zika virus infection among GBS registered cases (WHO 2016c). Based on the foregoing data, Zika fever, as a fast-emerging, pandemic-prone disease, was considered in the beginning of 2016 a Public Health Emergency of International Concern (WHO 2016d).

Originally restricted to Africa and the Indian subcontinent, chikungunya has recently been reported from Europe and the Americas (Chaves et al. 2012, WHO 2016e). In the American continent, cases of this fever, whose symptoms may persist for months, have been detected in 33 countries and territories since December 2013. In Brazil, from January to December 2015, a total of 22,813 cases, including three deaths, was informed (PAHO 2016d). Therefore, chikungunya, as well as dengue and Zika fevers, has emerged as a serious public health problem.

Despite research advances from the pharmaceutical industry, developing dengue, Zika and chikungunya vaccines has proven a complex task and these vector-borne diseases remain a global public health challenge. Meanwhile, controlling the incidence of dengue, Zika and chikungunya fevers has largely depended on effectively fighting its main primary vector, *Ae. aegypti*, a mosquito highly adapted to urban environments. So, strategies must be strengthened, particularly in the Americas, aiming at its elimination. Reducing the vector population with synthetic insecticides to target its larval stage in breeding sites remains the main strategy for successful vector management (Dusfour et al. 2011). Nonetheless, a major obstacle to this approach has been the continued use of conventional insecticides, such as organophosphates and carbamates - a practice that leads to development of resistant species (Koou 2014). In addition, the deleterious effects of synthetic insecticides on the environment and non-target organisms have stimulated the search for alternative eco-friendly larvicidal agents, such as plant-derived secondary metabolites (Geris et al. 2012, Garcez et al. 2013, Góis et al. 2013, Stappen et al. 2014, Anholeti et al. 2015, Bezerra-Silva et al. 2015). As part of our ongoing screening program for plants of the Cerrado and Pantanal - two outstanding biomes in Midwest Brazil - as potential sources of agents against *Ae. aegypti* (Garcez et al. 2009), a total of 36 crude extracts from different anatomical parts (aerial parts, leaves, stems, roots, fruits and/or seeds) of 27 species belonging to 18 different families were assessed for their larvicidal activity in the present work. Of the extracts evaluated, only that from seeds of *Guarea kunthiana* (Meliaceae) proved active, being therefore selected for a bioassay-guided fractionation. Herein, we report the isolation and structure elucidation of its bioactive constituent.

## MATERIALS AND METHODS

^1^H and ^13^C NMR spectra were obtained in CDCl_3_ (Cambridge Isotope Laboratories, Andover, USA) on a Bruker DPX-300 spectrometer (Karlhue, Germany), operating at 300.13 MHz (^1^H)/75.47 MHz (^13^C). EIMS data were acquired at 70 eV on a Shimadzu (Tokyo, Japan) QP5000 DI-50 instrument. Pre-coated silica gel GF254 chromatoplates (Merck, Darmstadt, Germany) were used for analytical thin-layer chromatographic procedures. Silica gel 60 (230-400 mesh, Merck) and silica gel 60 RP-18 (230-400 mesh, Merck) were used for column chromatography.

*Plant material* - The plants investigated (Table I) were collected from Cerrado and Pantanal areas in Mato Grosso do Sul, Brazil, from August 2009 to August 2012 and identified by Dr Arnildo Pott (Universidade Federal de Mato Grosso do Sul, Campo Grande, Brazil). Voucher specimens are deposited at the CGMS Herbarium of the Universidade Federal de Mato Grosso do Sul.

**TABLE I:** Plants evaluated and activity of their ethanol extracts against third-instar *Aedes aegypti* larvae

Species (Family)	Voucher number	Site of collection/Biome	Plant part	LC_50_ (mg/mL)
*Anona cornifolia* A. St.-Hil. (Annonaceae)	WG234	Campo Grande/Cerrado	Aerial parts	I
*Vernonia ferruginea* Less. (Asteraceae)	WG223	Campo Grande/Cerrado	Stems	I
*V. ferruginea* Less. (Asteraceae)		Campo Grande/Cerrado	Leaves	I
*Tabebuia aurea* (Silva Manso) Benth. & Hook. f. ex S. Moore (Bignoniaceae)	WG073	Corumbá/Pantanal	Leaves	I
*T. aurea* (Silva Manso) Benth. & Hook. f. ex S. Moore (Bignoniaceae)		Corumbá/Pantanal	Stems	I
*T. avellanedae* var. paulensis (Bignoniaceae)	WG150	Corumbá/Pantanal	Stems	I
*T. impetiginosa* (Mart. ex DC.) Standl. (Bignoniaceae)	WG177	Aquidauana/Cerrado	Leaves	I
*T. ochracea* (Cham.) Standl. (Bignoniaceae)	WG224	Campo Grande/Cerrado	Leaves	I
*Costus spiralis* (Jacq.) Roscoe (Costaceae)	WG215	Campo Grande/Cerrado	Aerial parts	I
*C. spiralis* (Jacq.) Roscoe (Costaceae)		Campo Grande/Cerrado	Roots	I
*Doliocarpus dentatus* (Aubl.) Standl. (Dilleniaceae)	WG248	Dois Irmãos do Buriti/Cerrado	Leaves	I
*Erythroxylum campestre* A. St.-Hil. (Erythroxylaceae)	WG226	Campo Grande/Cerrado	Leaves	I
*Cassytha filiformis* L. (Lauraceae)	WG072	Campo Grande/Cerrado	Leaves	I
*Mezilaurus crassiramea (Meisn.) Taub. ex Mez* (Lauraceae)	WG256	Corumbá/Pantanal	Fruits	I
*Ocotea suaveolens* (Meisn) Benth. & Hook. f. ex Hieron. (Lauraceae)	WG076	Corumbá/Pantanal	Fruits	I
*Strychnos pseudoquina* A. St.-Hil. (Loganiaceae)	WG108	Campo Grande/Cerrado	Fruits	I
*S. pseudoquina* A. St.-Hil. (Loganiaceae)		Campo Grande/Cerrado	Leaves	I
*S. pseudoquina* A. St.-Hil. (Loganiaceae)		Campo Grande/Cerrado	Stems	I
*Guazuma ulmifolia* Lam. (Malvaceae)	WG17	Campo Grande/Cerrado	Leaves	I
*Miconia albicans* (Sw.) Steud. (Melastomataceae)	WG181	Campo Grande/Cerrado	Stems	I
*Guarea kunthiana* A. Juss. (Meliaceae)	WG239	Bodoquena/Cerrado	Fruits (peel and pulp)	I
*G. kunthiana* A. Juss. (Meliaceae)		Bodoquena/Cerrado	Seeds	169.93
*Trichilia pallida* Sw. (Meliaceae)	WG232	Corumbá/Pantanal	Fruits	I
*Hyeronima alchorneoides* Allemão (Phyllanthaceae)	WG213	Campo Grande/Cerrado	Leaves	I
*Roupala montana* Aubl. (Proteaceae)	WG202	Aquidauana/Cerrado	Leaves	I
*R. montana* Aubl. (Proteaceae)		Aquidauana/Cerrado	Stems	I
*Palicourea marcgravii* A. St.-Hil. (Rubiaceae)	WG218	Campo Grande/Cerrado	Leaves	I
*P. marcgravii* A. St.-Hil. (Rubiaceae)		Campo Grande/Cerrado	Stems	I
*Pogonopus tubulosus* (A. Rich.) K. Schum. (Rubiaceae)	WG230	Aquidauana/Cerrado	Fruits	I
*Psychotria carthagenensis* Jacq. (Rubiaceae)	WG221	Campo Grande/Cerrado	Stems	I
*Chrysophyllum marginatum* (Hook. & Arn.) Radlk. (Sapotaceae)	WG219	Campo Grande/Cerrado	Stems	I
*Matayba guianensis* Aubl. (Sapindaceae)	WG093	Aquidauana/Cerrado	Leaves	I
*Siparuna guianensis* Aubl. (Siparunaceae)	WG220	Campo Grande/Cerrado	Fruits	I
*Cestrum virgatum Ruiz & Pav.* (Solanaceae)	WG231	Aquidauana/Cerrado	Stems	I
*Solanum lycocarpum* A. St.-Hil. (Solanaceae)	WG227	Campo Grande/Cerrado	Fruits	I
*S. lycocarpum* A. St.-Hil. (Solanaceae)		Campo Grande/Cerrado	Leaves	I

I: inactive (LC_50_ value > 1000 mg/mL)

*Preparation of extracts* - Air-dried and powdered plant material (aerial parts, leaves, stems, roots, fruits and/or seeds, at least 500 g each) was extracted with ethanol (4 x 2L) at room temperature. After concentration *in vacuo*, the residue obtained from each extract was stored at -18ºC until biological screening was performed.

*Bioassay-guided fractionation of seeds of G. kunthiana* - Fruits of *G. kunthiana* were collected in August 2012 and their seeds (1,000 g) were separated from the pulp and peel (3,000 g). The seeds were extracted with ethanol (4 x 4 L) for seven days and filtered. After concentration under reduced pressure, the bioactive ethanol extract was subsequently partitioned between MeOH-H_2_O (9:1) and hexane. Water was added to the hydromethanolic phase to yield a MeOH-H_2_O (1:1) mixture, which was partitioned with EtOAc. The hexane (9.0 g), EtOAc (14.0 g), and hydromethanolic (2.0 g) phases were tested against *Ae. aegypti* larvae, and the larvicidal activity was found to reside in the EtOAc solubles (LC_50_ = 105.70 mg/mL). An aliquot of this bioactive phase (11.0 g) was then chromatographed on an RP-18 silica gel (350.0 g) column, using step gradient elution with MeOH-H_2_O (4:6, 6:4, 8:2), MeOH, and CHCl_3_ to give five fractions (F1-F5) of 500 mL each. Testing for larvicidal activity showed fraction F3 (2.0 g), containing two major components, to be bioactive (LC_50_ = 15.20 mg/mL). An aliquot of this fraction (800.0 mg) was further subjected to column chromatography on silica gel (30.0 g) using CHCl_3_ (750 mL), CHCl_3_-MeOH (9.7:0.3, 750 mL), CHCl_3_-MeOH (9.5:0.5, 750 mL), CHCl_3_-MeOH (9.3:0.7, 750 mL), and CHCl_3_-MeOH (9:1, 750 mL), yielding protolimonoids **1** (510.0 mg, major compound, eluted with CHCl_3_-MeOH 9.5:0.5) and **2** (210.6 mg, eluted with CHCl_3_-MeOH 9.3:0.7), both of which were tested against *Ae. aegypti* larvae. Only compound **1** proved bioactive (LC_50_ = 14.44 mg/mL). Purity of protolimonoids **1** and **2** was established on the basis of their ^1^H and ^13^C NMR spectra and by chromatographic techniques.

*Melianodiol (* ***1*** *)* - colorless amorphous powder; EIMS (70 eV), *m/z* (rel. ab.): 488 (M^+.^, 4), 470 (8) [M - H_2_O], 455 (25) [M - ^.^CH_3_], 412 (17) [470 - CH_3_COCH_3_], 397 (59) [455 - CH_3_COCH_3_/ 412 - ^.^CH_3_], 379 (34) [ 397 - H_2_O], 365 (92) [397 - CH_3_OH], 297 (67) [379 - C_5_H_6_O], 59 (100) [ C_3_H_7_O^+^]; ^1^H NMR (CDCl_3_, 300 MHz): δ 2.76 (1H, td, *J* = 14.4, 5.2 Hz, 2-H); 5.25 (1H, brs, 7-H); 0.83 (3H, s, 18-H); 1.01 (3H, s, 19-H); 5.32 (1H, brs, 21-H); 4.38 (1H, m, 23-H); 3.15 (1H, brs, 24-H); 1.26 (3H, s, 26-H); 1.28 (3H, s, 27-H); 1.03 (3H, s, 28-H); 1.12 (3H, s, 29-H); 1.03 (3H, s, 30-H); ^13^C-NMR (CDCl_3_, 75 MHz): δ 38.5 (C-1); 35.0 (C-2); 216.9 (C-3); 47.8 (C-4); 52.3/52.4 (C-5); 24.3 (C-6); 118.0 (C-7); 145.6 (C-8); 48.3/48.8 (C-9); 35.1 (C-10); 17.6 (C-11); 30.1/31.4 (C-12); 43.5/43.6 (C-13); 50.7 (C-14); 34.2 (C-15); 27.2 (C-16); 45.3 (C-17); 21.5 (C-18); 12.7 (C-19); 45.2/46.3 (C-20); 96.7/102.2 (C-21); 30.2 (C-22); 78.7 (C-23); 74.8/75.8 (C-24); 73.2 (C-25); 26.5 (C-26); 26.7 (C-27); 24.5 (C-28); 23.2 (C-29); 27.4 (C-30). ^1^H and ^13^C NMR, and mass spectra of melianodiol are shown in Supplementary figures .

*Meliantriol* ***(2)*** - colorless amorphous powder; EIMS (70 eV), *m/z* (rel. ab.): 490 (M^+.^, 15), 475 (19) [M - ^.^CH_3_], 457 (40) [475 - H_2_O], 439 (61) [457 – H_2_O], 399 (39) [457 - CH_3_COCH_3_], 381 (40) [399 - H_2_O], 367 (100) [399 - CH_3_OH], 349 (20) [367- H_2_O], 281 (39) [ 381 - C_5_H_8_O_2_]; ^1^H NMR (CDCl_3_, 300 MHz): δ 3.22 (1H, dd, *J* = 11.0, 3.5 Hz, 3-H); 5.21 (1H, brs, 7-H); 0.82 (3H, s, 18-H); 0.72 (3H, s, 19-H); 5.24 (1H, brs, 21-H); 4.34 (1H, m, 23-H); 3.14 (1H, brs, 24-H); 1.26 (3H, s, 26-H); 1.24 (3H, s, 27-H); 0.84 (3H, s, 28-H); 0.94 (3H, s, 29-H); 0.96 (3H, s, 30-H); ^13^C-NMR (CDCl_3_, 75 MHz): δ 37.2 (C-1); 27.5 (C-2); 79.1 (C-3); 39.0 (C-4); 50.7 (C-5); 24.0 (C-6); 118.2 (C-7); 145.4 (C-8); 48.8 (C-9); 34.9 (C-10); 17.5 (C-11); 31.6 (C-12); 43.7 (C-13); 50.7 (C-14); 34.2 (C-15); 27.3 (C-16); 50.7 (C-17); 23.2 (C-18); 13.1 (C-19); 45.3/46.5 (C-20); 97.1/102.3 (C-21); 34.2 (C-22); 77.4/78.7 (C-23); 75.1 (C-24); 73.4 (C-25); 26.5 (C-26); 26.7 (C-27); 27.5 (C-28); 14.7 (C-29); 27.3 (C-30). ^1^H and ^13^C NMR, and mass spectra of meliantriol are shown in Supplementary figures.

*Larvicidal activity assay against Ae. aegypti* - The bioassays were performed according to World Health Organization protocols (WHO 1981) with slight modifications (Garcez et al. 2009). Mosquito eggs (Rockefeller strain) were obtained from a colony maintained at our laboratory. The adult colony was provided with 10% saccharose solution and adult female mosquitoes were blood-fed on Swiss mice for egg production. The eggs were then hatched by immersion in distilled water for 1 h, followed by incubation in the dark at 27 (± 2) ºC and 70 (± 5) % relative humidity. Larvae were fed on fish food and, upon reaching the third instar, the larvae were used in the bioassays. Stock solutions at 1,000, 500, 250, 125, 62.5 and 31.25 mg/mL were prepared from each plant extract, as well as from the partition phases and fractions originating from *G. kunthiana* seeds. To this end, the samples were dissolved in distilled water containing 0.5% dimethylsulfoxide (DMSO). For F3 (the bioactive fraction), serial diluting yielded test solutions of concentrations as low as 15 mg/mL. Compounds **1** and **2** were each initially tested at 100, 80, 60, 40 and 20 mg/mL. Compound **1** (the bioactive protolimonoid) was subsequently tested at concentrations as low as 15 mg/mL to allow calculating its LC_50_ value. The purity of the isolated compounds tested was ≥ 95%. Ten third-instar larvae were placed in a beaker containing 5 mL of test sample. For each concentration, 40 larvae were exposed (four replicates of 10 larvae each). A set of controls was also run, using 0.5% DMSO in distilled water in four replicates. Each assay was repeated three times at different days and showed no significant variation. Larval mortality was recorded 24 h after treatment at 27 (± 2) ºC, during which no food was given to the larvae. Larvae were considered dead when they did not respond to stimuli (gentle poking with a pipette). All the experiments were carried out at 27 (± 2) ºC and 70 (± 5) % relative humidity.

Considering the mortality of the larvae at the experimental concentrations, the LC_50_ and LC_90_ values (µg/mL), slope and Chi-square value were calculated using the Probit analysis on IBM SPSS Statistics 22.0. Comparison among samples in relation to the LC_50_ and LC_90_ values was performed using the 95% confidence interval from LCs ratio (Wheeler et al. 2006).

Mosquito eggs (Rockefeller strain) were obtained from a colony maintained at our laboratory (permit 469/2012 from the Animal Ethics Committee of the Universidade Federal de Mato Grosso do Sul), according to the Conselho Nacional de Controle de Experimentação Animal - CONCEA/Ministério da Ciência, Tecnologia e Inovação-MCTI (http://www.mct.gov.br/index.php/content/view/310553.html). The authors declare no conflicts of interest.

## RESULTS

Of the 36 crude extracts tested, only that prepared from *G. kunthiana* seeds proved active against *Ae. aegypti* larvae (LC_50_ = 169.93 μg/mL). No larval deaths occurred with any of the other extracts, not even that prepared from *G. kunthiana* fruit peel and pulp, which had LC_50_ values higher than 1,000 μg/mL (Table I).

Based on these results, a bioassay-guided investigation of the ethanol extract of *G. kunthiana* seeds was conducted, and the larvicidal activity was found to reside in the EtOAc phase - with LC_50_ and LC_90_ values of 105.70 and 408.91 mg/mL, respectively (Table II), obtained by extract partitioning, since both hexane and hydromethanolic phases exhibited LC_50_ values higher than 1,000 µg/mL.

**TABLE II:** Toxicity of extract, fractions and isolated compound against 3rd instars of *Aedes aegypti* after 24 h exposure

Treatment	LC_50_ (μg/mL) (95% FL)	LC_90_ (μg/mL) (95% FL)	Slope (±SE)	*x* ^2^
*Guarea kunthiana* EtOH extract	169.93a (141.62-203.41)	496.11a (389.56-692.46)	2.75±0.29	8.58
EtOAc phase	105.70b (83.85-130.30)	408.91b (310.56-598.17)	2.18±0.24	24.88
Bioactive fraction	15.20c (14.59-15.62)	18.25c (17.80-18.91)	16.11±2.03	12.69
Melianodiol (**1**)	14.44d (13.53-15.01)	17.54d (17.10-18.15)	15.20±2.27	14.37
Meliantriol (**2**)	> 100	> 100	-	-

FL = fiducial limits; SE = standard error. Values within a column followed by different letters are significantly different (p < 0.05) based on the ratio test of 95% fiducial limits.

This phase was then subjected to column chromatography procedures and the resulting bioactive fraction (LC_50_ = 15.20 mg/mL, Table II), containing two principal components, was further separated, leading to the isolation and characterisation of the known protolimonoids melianodiol (**1**, major compound) and meliantriol (**2**) (Figure). Their structures were established on the basis of ^1^H and ^13^C NMR spectroscopic techniques supported by mass spectra, as well as by comparison with previously reported data, and authentic samples (Puripattanavong et al. 2000, Kurimoto et al. 2014, Miguita et al. 2015).

**Figure:**
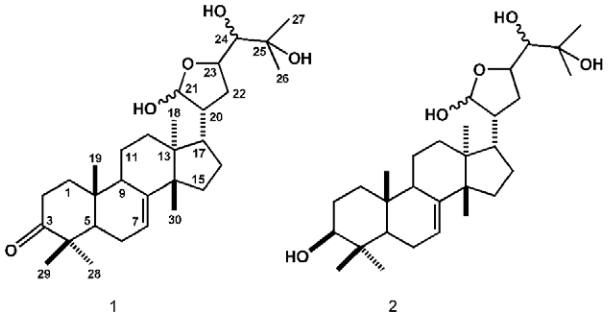
Structures of protolimonoids melianodiol (1) and meliantriol (2).

After evaluation for toxicity against *Ae. aegypti* larvae, only compound **1** proved biologically active, with LC_50_ and LC_90_ values of 14.44 and 17.54 mg/mL, respectively, as depicted in Table II, while **2** had an LC_50_ value above 100 mg/mL.

All samples showed dose/response curve (slope) different from 0 degree (p < 0.001) and *p* values greater than 0.15 on chi-square test (p = 0.995; p = 0.303; p = 1.000; p = 1.000, respectively). All samples were also significantly different with respect to the LC_50_ and LC_90_ values (based on ratio test; p < 0.05), and also presented decreasing LC values from crude extract to the isolated bioactive compound (melianodiol, **1**).

## DISCUSSION

In the present study, the protolimonoid melianodiol (**1**) was isolated as the larvicidal constituent of the bioactive ethyl acetate phase obtained after partitioning of the ethanol extract of the seeds of *G. kunthiana*. Meliantriol (**2**), which was also obtained from the bioactive fraction, was nevertheless devoid of any larval toxicity, even at the highest concentration tested. These results indicate that the larvicidal activity of **1** stems from the presence of the carbonyl moiety at C-3 in the 21,23-epoxy-21,24,25-trihydroxy-tirucall-7-ene-type skeleton, since its corresponding reduced derivative **2**, which differs from **1** only for bearing a hydroxyl function at that same carbon, was found to be inactive.

Meliaceae, the family to which the genus *Guarea* belongs, is a rich source of secondary metabolites, including limonoids and protolimonoids (Taylor 1984, Tan & Luo 2011). In a previous investigation of *G. kunthiana*, we isolated **1** and **2** from the fruits, and di- and sesquiterpenes, poliprenol-12, and α- and d-tocopherols from the leaves (Garcez et al. 2004, Miguita et al. 2015). The fruits also yielded the limonoids humilinolide E, 2-hydroxy-6-deoxyswietenine, swietenin acetate and methyl angolensate (Miguita et al. 2014), none of which were found to be present in our *G. kunthiana* bioactive fraction from which melianodiol (**1**) was obtained, despite the fact that limonoids are known for their biological, and particularly insecticidal, properties (Tan & Luo 2011). They were shown to be present, however, in a more polar, otherwise inactive fraction resulting from chromatographic separation of the bioactive EtOAc phase. To our knowledge, only the protolimonoid nilocitin has hitherto been reported to exhibit significant growth disruption and morphological deformities against *Ae. aegypti* larvae and pupae (Reegan et al. 2014), and the present investigation is therefore the first to isolate melianodiol by conduction a bioassay-guided study of plant constituents active against *Ae. aegypti* larvae.

The foregoing results revealed melianodiol to be a potential candidate for the development of an ecologically sound alternative to environmentally hazardous synthetic insecticides currently employed to control *Ae. aegypti* larvae in areas where dengue, Zika and chikungunya fevers are of concern. Also, melianodiol was isolated from seeds, a feature that encourages the conservation and sustainable use of *G. kunthiana* as a source of this bioactive compound. The findings warrant investigating other Meliaceous species, as well as members of the Simaroubaceae and Rutaceae, from which protolimonoids have also been obtained, for their potential towards the development of new biocontrol agents against *Ae. aegypti* larvae.
