# Supplementary material for: Larvicidal efficacies of plants from Midwestern Brazil: melianodiol from Guarea kunthiana as a potential biopesticide against Aedes aegypti
Source: Mem Inst Oswaldo Cruz. 2016 Jun 13;111(7):469–74. doi: 10.1590/0074-02760160134 (PMC4957500; doi:10.1590/0074-02760160134)
Supplement: Supplementary file 1 [file 0074-0276-mioc-0074-02760160134-sd.pdf]

NMR and mass spectral data of melianodiol and meliantriol.

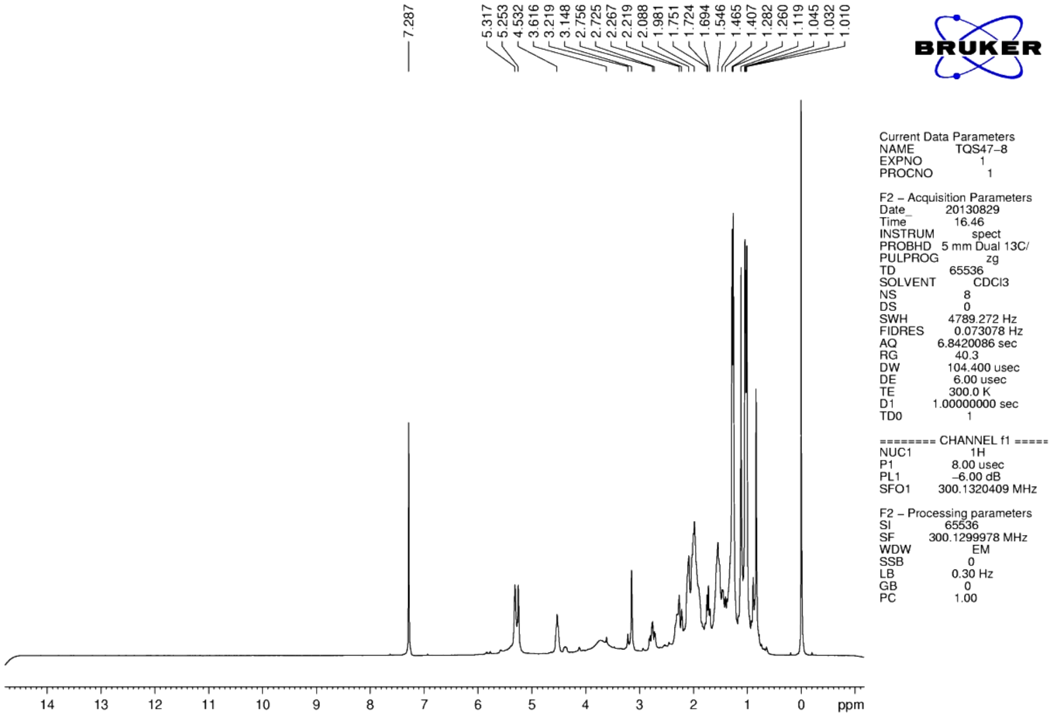

^{1}H NMR spectrum of melianodiol (**1**) (300 MHz - $CDCl_3$).

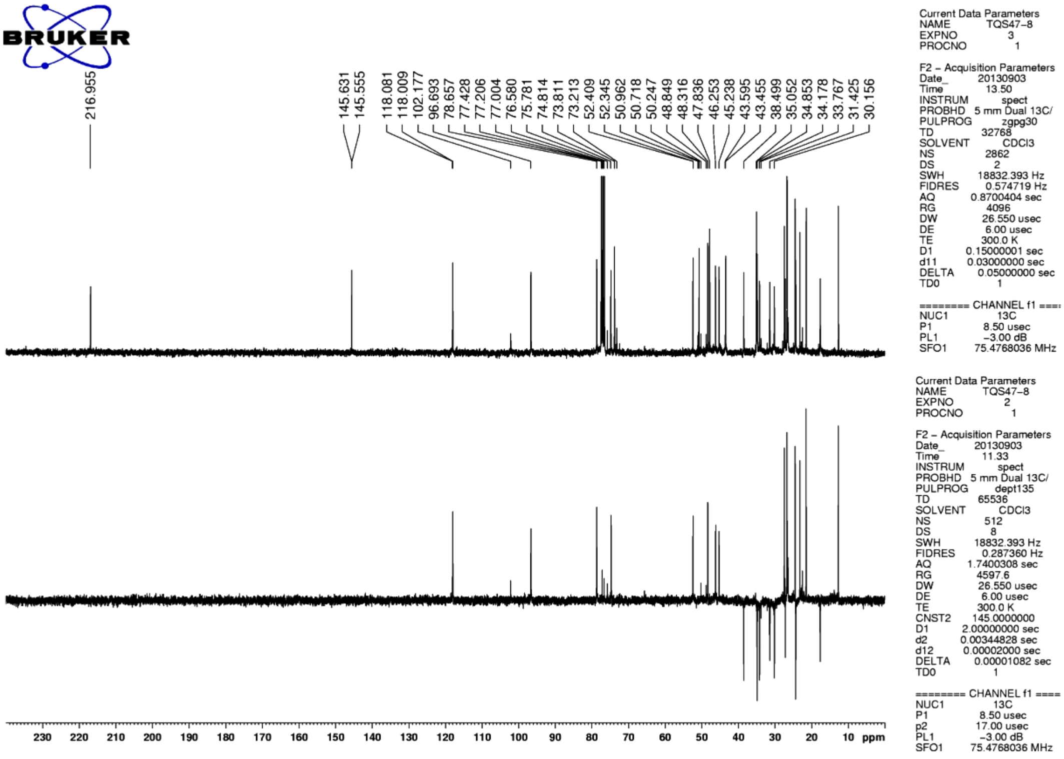

^{13}C NMR spectra of melianodiol (**1**) (BBD and DEPT 135) (75 MHz - $CDCl_3$).

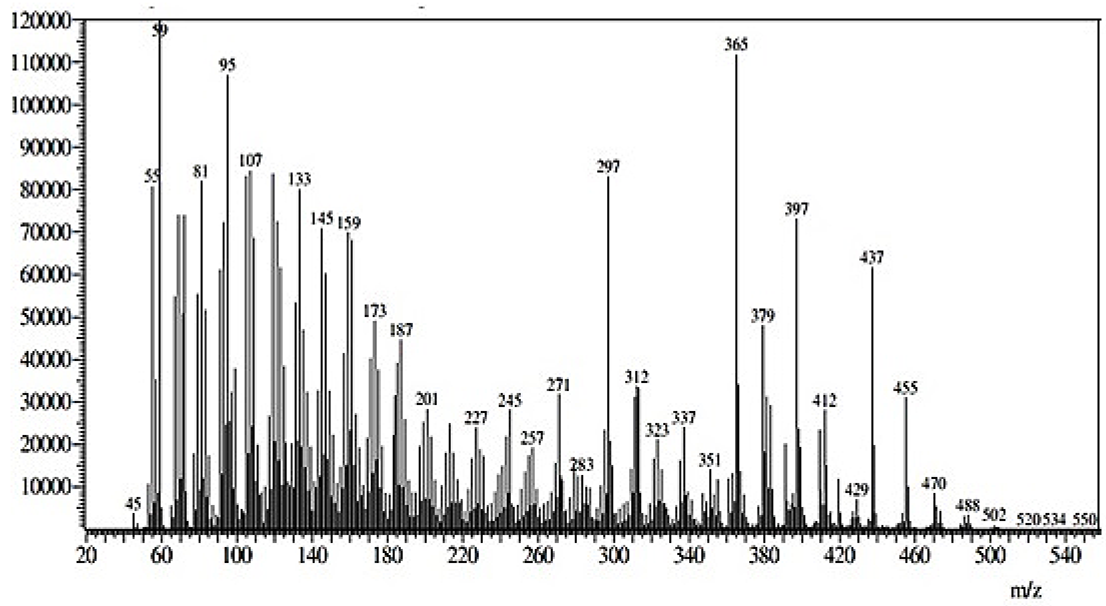

EIMS (70 eV) of melianodiol (**1**).

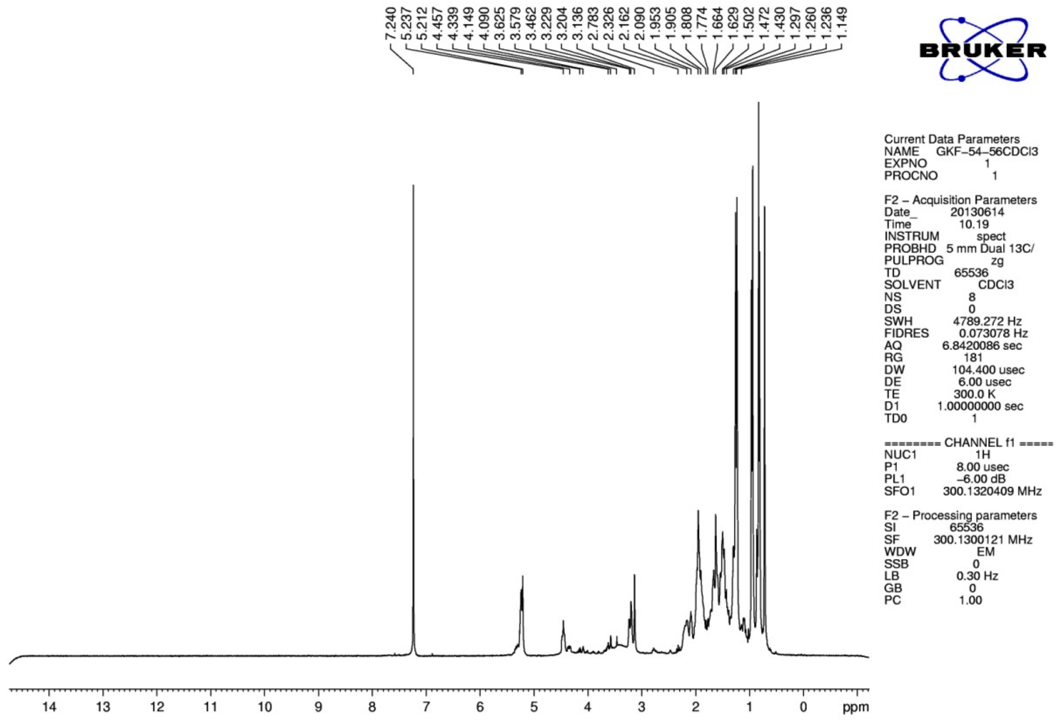

^{1}H NMR spectrum of meliantriol (**2**) (300 MHz - $CDCl_3$).

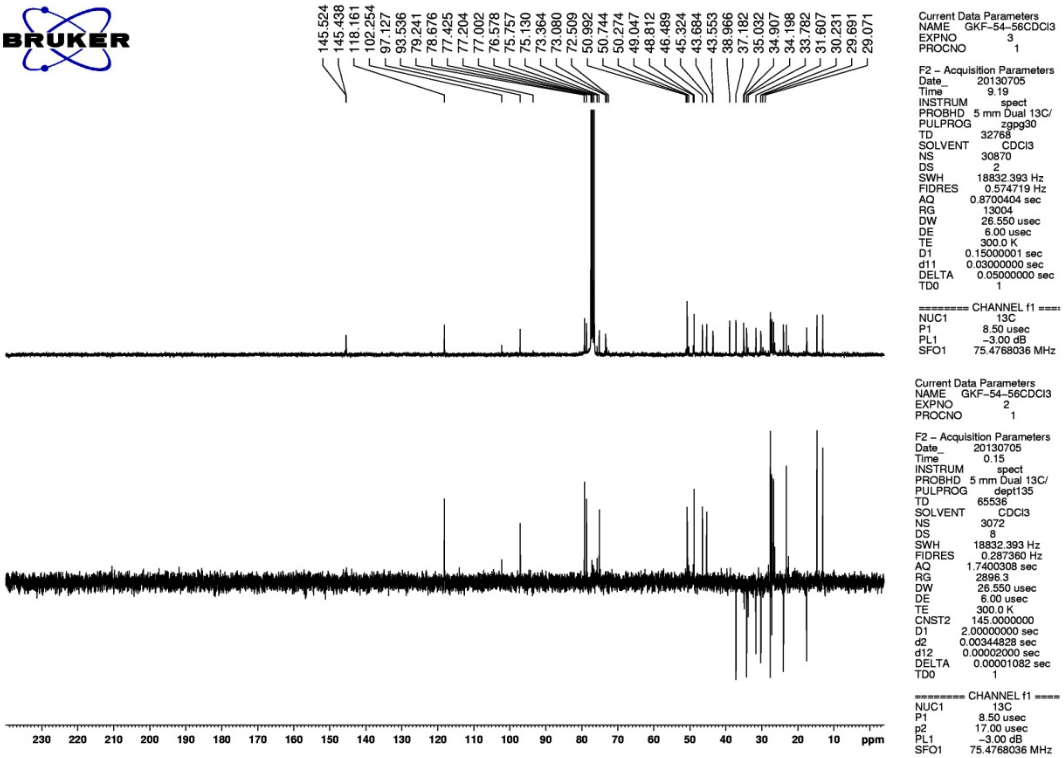

^{13}C NMR spectra of meliantriol (**2**) (BBD and DEPT 135) (75 MHz - $CDCl_3$).

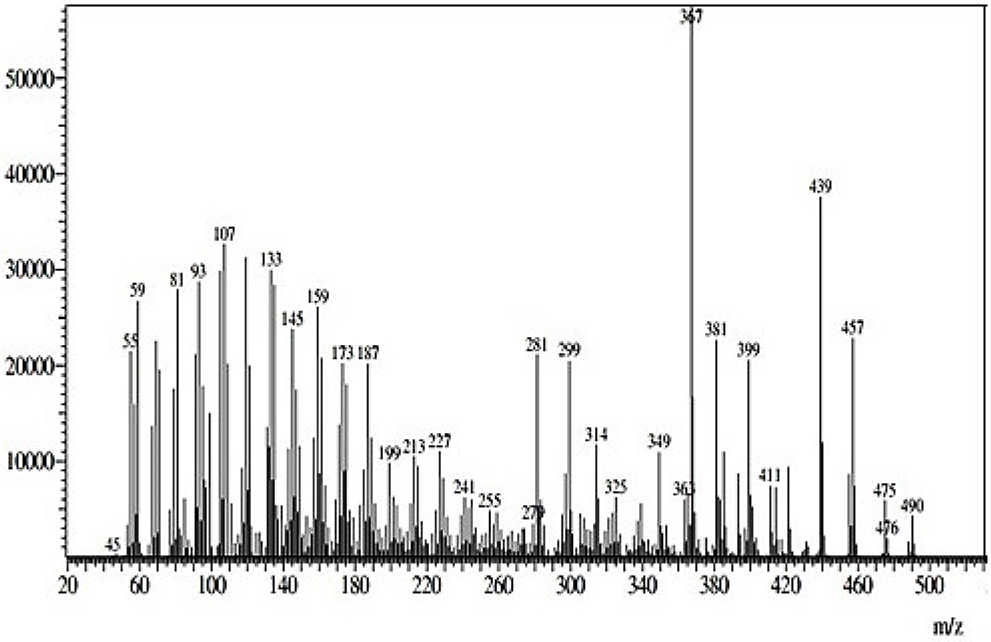

EIMS (70 eV) of meliantriol (**2**).
